# Assessment of community psycho-behavioral responses during the outbreak of novel coronavirus (2019-nCoV): a cross-sectional study

**DOI:** 10.3934/publichealth.2022003

**Published:** 2021-11-09

**Authors:** Doaa M Magdy, Ahmed Metwally, Omar Magdy

**Affiliations:** 1 Department of Chest Diseases, Faculty of Medicine, Assuit University, Egypt; 2 Medical student Faculty of Medicine, Assuit University, Egypt

**Keywords:** anxiety, coronavirus, precaution, psychological impact

## Abstract

The novel coronavirus 2019 (2019-nCoV) is a public health emergency of international concern resulting in adverse psychological impacts during the epidemic.

**Methods:**

From 1 to 10 April 2020, we conducted an online survey. The online survey collected information on demographic data, precautionary measures against (2019-nCoV), self-health evaluation, knowledge, and concerns about (2019-nCoV), and appraisal of crisis management. The psychological impact was assessed by the General Anxiety Disorder 7-item (GAD-7) scale. The designed questionnaire was answered by participants, and collected data were statistically analyzed.

**Results:**

This study included 1200 respondents. In total, 80% of respondents rated the psychological impact; 18% reported minimal anxiety; 34% reported mild anxiety, and 48% with moderate anxiety symptoms. A large proportion (74%) believed that they were very or somewhat likely to contract (2019-nCoV) while only (35%) believed they were unlikely to survive if they contracted the disease. (58%) of the respondents, practiced the precautionary measures directed against person-to-person droplet spread. Respondents with a moderate level of anxiety were most likely to take comprehensive precautionary measures against the infection. Also, older, female, more educated people as well as those who are more likely to contract the infection.

**Conclusions:**

During the outbreak, more than half of the respondents rated the psychological impact as moderate anxiety. Thus, the psychological needs during the outbreak should be addressed appropriately. Our results highlight the need to promote protective personal health practices to interrupt the transmission of the (2019-nCoV) in the community. Therefore, educational public programs about preventive measures should be targeted at the identified groups with low current uptake of precautions.

## 1. Background

The novel coronavirus disease 2019 (2019-nCoV) is a global health crisis and is by far the largest outbreak of atypical pneumonia since the severe acute respiratory syndrome (SARS) epidemic in 2003 [1]. (2019-nCoV) was first identified in December 2019 in China and had spread to 13 countries by January 24, 2020 [2].

On 30 January 2020, the World Health Organization (WHO) declared the novel acute respiratory syndrome coronavirus to be a Public Health Emergency of International Concern. (2019-nCoV) is spreading from person to person primarily via direct contact or through direct droplets spread by coughing or sneezing from an infected individual. As a result, WHO recommended that health authorities should raise awareness to limit the spread of respiratory infections [3].

It has been estimated that the median incubation period of the virus is 5.1 days. The average basic reproductive number and doubling time were estimated to be 3.28 and 2.5 days respectively [4],[5]. It has been reported that more than 80% of infected individuals are asymptomatic or show mild clinical symptoms, 15% develop more severe symptoms, and 5% become critically ill. The case fatality rate is estimated at 2–3% [5].

By the end of February, the virus had spread to countries worldwide [5], so that on March 8, Egypt announced that a 60-year-old German tourist died in the touristic town Hurghada, Egypt, and this was the first German fatality from the virus. The escalation in the number of cases by the middle of March prompted the government to take more strict arrangements and encouraged the public to become accustomed to hand hygiene and social distancing [7].

By 25 May 2020, Egypt recorded 17,967 cases of (2019-nCoV), including 783 deaths. Egypt had the highest risk of (2019-nCoV) infection importation among African countries [8].

Since the announcement of the first case, Egypt released a coexistence plan to deal with the coronavirus pandemic, to reduce the number of infections and deaths. The government started multidisciplinary national coordination between different ministries. Egypt suspended schools and universities and encouraged electronic distance learning. Also, Egypt imposed a curfew from 7 PM until 6 AM, during which all restaurants, shopping centres and markets are closed from 5 PM to 6 PM and are subjected to a complete shutdown on Fridays and Saturdays. Besides, all means of public and private transportation are suspended during curfew hours. All international passenger flights were also suspended in an attempt to reduce the risk of importing infection. All sports and entertainment facilities were also banned to prevent the spread of (2019-nCoV) [8].

Egypt has reported 316,711 confirmed cases with 17,884 deaths with a case fatality rate of 2.2% [9]. Although the case fatality rate was low compared to other developed countries like USA or UK, people were anxious as the virus spread rapidly from one person to another through direct or indirect contact [10].

A pandemic is a public life-threatening condition disrupting social life, family life, occupational life, education, transportation, travelling, and other aspects of everyday life. Moreover, during the outbreak, a psychological impact is imposed on the general population. Anxiety and fear are common psychological responses to this frightening condition [11]. Fear of (2019-nCoV) was positively associated with depressive and anxiety symptoms' severity [12],[13]. Major stressors such as long duration of quarantine, fears for infection, inadequate information, stigma, or financial loss were related to an increased risk of anxiety [14].

Therefore, the purpose of this study, to examine four areas of public reaction to the (2019-nCoV) outbreak: preventive practices, perception of self-health, knowledge of (2019-nCoV), and appraisal of (2019-nCoV) crisis management.

## 2. Materials and methods

### 2.1. Study design and subjects' selection

We modified and expanded a specially designed questionnaire based on the author's experience and previously published studies on pandemics via an online survey [15],[16]. This survey was distributed through social media (e.g. Facebook, LinkedIn) to minimize personal contact during the outbreak between 1st April and 12th April 2020. The survey members of the general population were invited to join the study voluntarily.

The participants accessing the survey platform were informed about the nature of the research and data usage. Before entering the survey, respondents were requested to indicate their consent by ticking the consent checkbox, as a required field.

The questionnaires were filled out anonymously to protect participants' personal data. To secure anonymity, the setting “anonymize responses” was designed on the online platform; to protect the privacy of participants while collecting, analyzing, and reporting data. Ethical approval was received from Faculty of Medicine Ethics Committee, Assiut University before data collection. Consequently, a pilot test was conducted involving 30 people of different ages, sex, and educational levels.

Conciseness was achieved through the removal of redundant and irrelevant queries that could bring confusion. Questions were placed in a logical sequence to provide a better mental roadmap while filling out the survey. The time required for survey completion was estimated at around 15 min. Data collection took place over two weeks during the outbreak of (2019-nCoV).

### 2.2. Data collection

The questionnaire includes (1) basic sociodemographic data: sex, age, education level, marital, and employment status.

The authors included additional questions related to the (2019-nCoV) outbreak.

The structured questionnaire considered the following questions: (2) precautionary measures against (2019-nCoV) in the past 14 days (3) self-health evaluation and the psychological impact during (2019-nCoV) outbreak; (4) knowledge and concerns about (2019-nCoV); and (5) appraisal of crisis management.

#### 2.2.1. I-preventive measures

In the first part of the survey, we assessed the precautionary measures against (2019-nCoV) through questions with five answer choices, categorized from (Don't know to always), regarding how often the following behaviors were identified: covering mouth when coughing or sneezing; washing hands with soap and water or liquid hand- gel; washing hands immediately after coughing, or sneezing; wearing a mask in public areas; avoiding sharing utensils during meals, and washing hands after touching objects.

We constructed a composite index indicating the total number (from 0 to 8) of preventive measures taken. A dichotomous indicator of preventive behavior was calculated based on the mean number of precautions taken (4.65): “low” (<5) versus “high” (>6).

#### 2.2.2. II-self-health perception

The next part of the survey composed of 3 sets dealt with the respondents' perception of their health.

The first set covered seven physical health complaints over the previous 2 weeks including (Persistent high fever of 38°C, sore throat, running nose, having aches all over the body, headache, cough, rapid breathing). We created a composite index of symptoms by adding all instances of health complaints.

The second set of the survey assessed the level of anxiety using the General Anxiety Disorder 7-item (GAD-7) scale [17]. It is a 7-item questionnaire was developed that asked patients how often, during the last 2 weeks, they were bothered by each symptom. Response options were “not at all”, “several days”, “more than half the days” and “nearly every day”, scored as 0, 1, 2, and 3, respectively. The total anxiety score was divided into (0–4) – minimal anxiety, (5–9) – mild anxiety, (10–14) – moderate anxiety, and >14 – severe anxiety). The scale had an Alpha reliability coefficient of 0.824.

The third set addressed respondents' risk perception in terms of their likelihood of contracting (2019-nCoV) and survival if diagnosed with the disease. Scores were 4 (very likely) to 0 (don't know). Based on the average score (3.4; standard deviation [SD] 0.11), we created a dichotomous variable to contrast respondents who believed they were susceptible to contracting (2019-nCoV) (scores 3 and 4) with those who did not (scores 0–2).

#### 2.2.3. III-knowledge of (2019-nCoV)

We also administered basic questions on (2019-nCoV) mode of transmission (e.g. transmitted through saliva droplet, or airborne transmission, or hand contact. Responses were scored 0 (incorrect) or 1 (correct); a composite index indicated the number of correct answers, from none correct (0) to all three correct (3).

Also, we asked about various sources of this information; Internet; social media as Facebook and What's app, WHO websites, official statements by radio and television, family member, or others.

#### 2.2.4. IV-appraisal of crisis management

We addressed respondents' appraisal of crisis management. Five questions were carried out (Alpha reliability 0.813) to assess opinions on information distribution. Scores were 1 (very negative) to 6 (very positive). Based on the mean score (4.83; SD 0.617), we calculated a dichotomous index: negative appraisal (scores <4.7) versus positive appraisal (scores >4.8).

Finally, we evaluated the public's acceptance of quarantine regulations. The scores were dichotomized into “agreement” (1) versus “no agreement” and “don't know” (2).

#### 2.2.5. Procedures

All participants answered questions in order. A short introduction was provided that their identity would be kept confidential and all the collected information would be anonymized. Further, the respondents were made aware that participation in the study was voluntary, and that they could stop the interview at any time. Respondents were asked to click on the circle corresponding. Despite all of these measures, it is hard to assert that there was no bias.

#### 2.2.6. Statistical analysis

All statistical analyses were conducted using SPSS, version 21 (IBM Inc., Armonk, NY, USA). Quantitative measurement was expressed by mean (SD) and qualitative variables were presented as absolute frequency and percentage. Descriptive analysis; frequency (percentages), mean and standard deviation were used to examine the four factors ((nCoV-2019) prevention, perception of self-health, knowledge of (nCoV-2019), and perception of health authorities' crisis management).

Logistic regression was used to calculate the odds ratio (OR) with a 95% confidence interval to identify predictors for greater adoption of the recommended precautionary measures (defined as at least five of the eight specified strategies). P values of less than 0.05 were regarded as statistically significant.

## 3. Results

This survey was distributed through social media, including Facebook, by far the most popular social networking site in Egypt. We received responses from 1500 respondents. Out of them, 1200 (80%) respondents completed the survey, 200 (13.3%) did not complete the survey, and another 100 (6.7%) did not respond.

Sociodemographic characteristics of the study population are presented in Table 1. The majority of respondents were women (68%), Respondents were classified into five age categories: 18–24 (28%), 25–34 (52%), 35–44 (8%), 45–59 (6%), 60 years and above (6%). Overall, 64% were married, and 42% had at least 1 child. In total, 13.7% had no educational qualifications, 53.3% had high school diplomas, and 33% had university degrees.

**Figure 1.**
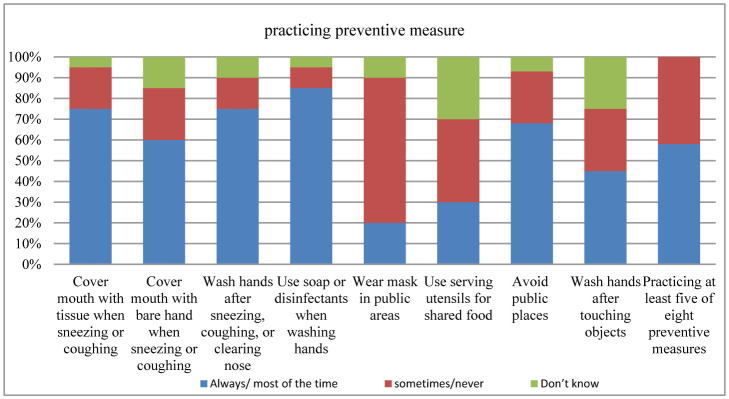
Practice of preventive measures to prevent transmission and contracting (2019-nCoV).

Figure 1 displays the respective proportions of respondents who reported practicing each of the specified precautionary measures as recommended to prevent the transmission and contracting of (2019-nCoV). It was observed that recommended preventive measures were not practiced uniformly. The most practiced measures in the last 2 weeks before the interview was using soap and disinfectants when washing hands (85%) and washing hands after sneezing, coughing, or clearing the nose (75%). The least practiced measure was wearing a mask over the mouth in public areas. A total of 20% wears masks, and most did so only when visiting a clinic or hospital or when the mask was part of a uniform (as in healthcare workers). Lastly, (58%) of the respondents practiced at least five of the eight specified preventive strategies to improve personal hygiene Table 2.

**Table 1.** Demographic characteristics of the study population.

	Sociodemographic factors	Number of respondents N (%)
Age in years		
	18–24	336 (28%)
	25–34	624 (52%)
	35–44	96 (8%)
	45–59	72 (6%)
	>60	72 (6%)
Marital status		
	Single	360 (30%)
	Married	768 (64%)
	Divorced/Separated	72 (6%)
Educational level		
	High school diplomas	640 (53.3%)
	University degrees	396 (33%)
	No educational qualifications	164 (13.7%)
Employment status		
	Unemployed	62 (5.2%)
	Farmers	20 (1.7%)
	Retired	6 (0.5%)
	Student	620 (51.7%)
	Employed	492 (41%)

Figure 1 displays the respective proportions of respondents who reported practicing each of the specified precautionary measures as recommended to prevent the transmission and contracting of (2019-nCoV). It was observed that recommended preventive measures were not practiced uniformly. The most practiced measures in the last 2 weeks before the interview was using soap and disinfectants when washing hands (85%) and washing hands after sneezing, coughing, or clearing the nose (75%). The least practiced measure was wearing a mask over the mouth in public areas. A total of 20% wears masks, and most did so only when visiting a clinic or hospital or when the mask was part of a uniform (as in healthcare workers). Lastly, (58%) of the respondents practiced at least five of the eight specified preventive strategies to improve personal hygiene Table 2.

**Table 2.** Variables used in the analysis of public reaction during (2019–nCoV) outbreak.

	%	Mean	SD
**I-Practice of preventive measures**		4.65	1.62
**Practicing each of eight measures during the past 14 days.**			
Cover mouth with a tissue when sneezing or coughing	75		
Cover mouth with bare hand when sneezing or coughing	60		
Wash hands after sneezing, coughing, or clearing nose	75		
Use soap or disinfectants when washing hands	85		
Wear a mask in public areas	20		
Use serving utensils for shared food	30		
Avoid public places (e.g. restaurants)	68		
Wash hands after touching objects	45		
**Preventive measures taken over past 3 days (score 0–8)**			
5 or fewer of the eight measures	58		
6 or more of the eight measures	42		
**II-Symptoms (0–7) over the past 2 weeks**		0.46	0.81
None	77.6		
One or more	22.4		
**Anxiety level**		7.43	3.18
Minimal (0–4)	18		
Mild anxiety (5–9)	34		
Moderate anxiety (10–14)	48		
**Perceived likelihood of contracting (2019–nCoV)**		3.43	0.11
Very likely (4)	44		
Likely (3)	30		
Not very likely (2)	20		
Not likely at all (1)	3		
Don't know (0)	3		
**Likelihood of surviving if infected**		2.41	0.13
Very likely (4)	30		
Likely (3)	35		
Not very likely (2)	6		
Not likely at all (1)	4		
Don't know (0)	25		
**Knowledge of (2019-nCoV)**		1.72	0.92
No knowledge (0 of 3 answers correct) (0)	11.7		
1 of 3 answers correct (1)	25.0		
2 of 3 answers correct (2)	42.5		
3 of 3 answers correct (3)	20.7		
**Appraisal of crisis management**		4.73	0.61
“Strongly agree” and “Agree” that information by health authorities is:			
Accurate	82.2		
Sufficient	84.5		
Timely	84.4		
Trustworthy	87.8		
**Agreeable to 14–day quarantine after non close contact with (2019-nCoV) infected person and no symptoms**		2.1	0.12
Agree	68		
Don't agree	24		
Don't know	8		

Respondents' perception of their health was generally positive. We reported the mean number of health complaints in our sample was 0.46 (SD 0.81). A relatively lower proportion of respondents (22.4%) reported having any of our seven physical health complaints over the previous 2 weeks.

It was observed that the mean anxiety score was 7.43 (SD 3.18). The survey showed a moderate anxiety level among respondents; only 18% of respondents reported minimal anxiety. Most respondents (74%) believed that they were “very likely” or “somewhat likely” to contract (2019-nCoV) during the current outbreak. Those who thought they were likely to get the disease reported slightly more anxiety. Regarding the likelihood of surviving if they contracted the disease, 10% believed they were unlikely (6% “not very likely” and 4 % “not likely at all”) to survive but (25%) were uncertain about this.

**Figure 2.**
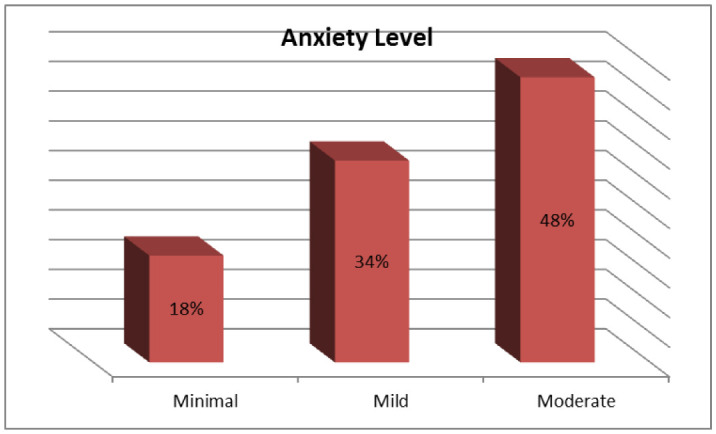
GAD-7 anxiety level category.

Figure 2: showed the percentages of the GAD-7 anxiety levels category among surveyed respondents. Out of the 1200 respondents, 216 (18%) reported minimal anxiety, 408 (34%) reported mild anxiety, 576 (48%) reported moderate anxiety, and none of them reported severe anxiety.

Regarding knowledge of (2019-nCoV), the sample correctly answered an average of 1.72 (SD 0.92) of 3 questions on (2019-nCoV) transmission. Approximately 63% answered two or more questions correctly; 11.7% did not answer any questions correctly.

**Figure 3.**
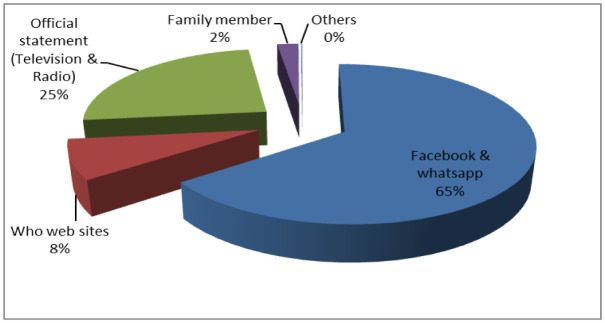
Information accessed resources.

Figure 3: showed the percentages of different information resources among respondents.

Internet and social networks, mainly Facebook and WhatsApp were the most common source of information (73.2%) followed by official statements or press releases by radio and television from MOHP (24.5). While family members and other resources were (2.3%).

Respondents had a generally high opinion of authorities' crisis management. More than 80% thought official information was accurate, clear, sufficient, timely, and trustworthy, and 68% were prepared to accept 14-day quarantine, even in the absence of symptoms or none -close contact with the (2019-nCoV) infected patient.

**Table 3.** Predictors for greater adoption of precautionary measures against (2019-nCoV) in the previous two weeks.

	Adjusted OR	95% CI	P-value
Anxiety level			
• Minimal or Mild (score <7.4)	1	Ref.	
• Moderate or severe (score >7.4)	2.51	1.62 to 3.04	=0.005
Gender			
• Men	1	Ref.	
• Women	1.77	1.12 to 2.46	=0.012
Age in years			
• <35	1	Ref.	
• >35	1.56	1.08 to 2.21	=0.039
Educational level			
• No educational qualifications	1	Ref.	
• University degrees.	1.27	1.05 to 2.96	=0.025
• High school diplomas	1.42	1.23 to 2.99	=0.009
Personal health evaluation			
Symptoms over past 2 weeks			
• None	1	Ref.	
• One or more	1.05	0.85 to 1.89	=0.619
Perceived likelihood of (2019-nCoV)			
• Not likely	1	Ref.	
• Likely	1.87	1.18 to 3.25	=0.036
Knowledge of (2019-nCoV)			
• Two or fewer correct answers	1	Ref.	
• Three correct answers	1.02	0.67 to 2.12	=0.814
Appraisal of crisis management			
Quality of official information			
• Below average (negative)	1	Ref.	
• Above average (positive)	1.22	0.94 to 1.84	=0.2
Agreeable to 14-day quarantine after non close contact with (nCoV-2019) infected person and no symptoms			
• Agree	1	Ref.	
• Don't agree/Don't know	1.13	0.79 to 1.9	=0.369

*Note: OR, odds ratio; CI, confidence intervals. * p,0.05, **p,0.01; _Odds ratios were adjusted for all other variables in the models.

Table 3 demonstrated the predictors for the adoption of precautionary measures among the study population. The final model contained five predictors after adjusting all other factors. It was noted that respondents who had a moderate level of anxiety were most likely to adopt more precautionary measures against (2019-nCoV) (OR 2.51; 95% CI 1.62 to 3.04, p < 001). Women were more likely to adopt comprehensive precautionary measures against (2019-nCoV) (OR 1.77; 95% CI 1.12 to 2.46, p < 001). Also, respondents aged >35 years were more inclined to take preventive measures (OR 1.56; 95% CI 1.08 to 2.21, p < 001) than their younger counterparts. Moreover, there was a strong positive dose-response gradient with an increasing level of educational attainment i.e. those with a university degree and higher school diploma were more likely to adopt comprehensive precautionary measures against (2019-nCoV) (OR 1.27; 95% CI 1.05 to 2.96, and 1.42; 95% CI 1.23 to 2.99, p < 001, respectively). Additionally, risk perception in terms of a higher self-perceived likelihood of contracting (2019-nCoV) was also a significant positive predictor for the adaption of preventive measures (OR 1.87; 95% CI 1.18 to 3.25, p < 001).

## 4. Discussion

The (2019-nCoV) outbreak constitutes one of the most challenging threats to a national and international public health concern. The epidemic had a significant impact on psychological healthcare communities. Therefore, the present study was designed to investigate the public reaction to the (2019-nCoV) outbreak: preventive practices, perception of self-health, knowledge of (2019-nCoV), and appraisal of crisis management among Egyptian adults during the period of the pandemic. Since the virus is in the ongoing spread, and the numbers of cases and fatalities are increasing, we hypothesized that the pandemic will cause negative psychological impact with more anxiety among the general population.

The fear of viral transmission during a pandemic is associated with precautionary measures, as well as avoidant behaviors to prevent infection. Preventive measures among others including; frequent hand hygiene, avoiding touching the face, wearing a medical mask in case of respiratory symptoms and social distancing.

This study explored that more than half of the respondents follow infection control protocols; wash their hands with soaps after touching contaminated objects, covered their mouth when coughing or sneezing, and wear masks. The experiences of the 2003 SARS-CoV epidemic could have changed the perception of the general public towards precautionary measures and have led to a positive effect by giving respondents a sense of confidence in prevention control measures [18].

In this study, the respondents rated their psychological impact during the outbreak; (48%) of them reported moderate anxiety symptoms. In accordance, Wang et al. [19] evaluated psychological impacts, depression, stress, and anxiety at the beginning of the (2019-nCoV) outbreak as measured by the DASS-21. The authors revealed that 53.8% experienced severe psychological impacts. Moreover, 16.5%, 28.8%, and 8.1% of the respondents reported moderate to severe levels of depression, anxiety, and stress [19]. Also, Xu et al. demonstrated that anxiety and fear were very prevalent among patients with (2019-nCoV) [20]. In another cross-sectional study, Al-Rabiaah et al. studied the impacts of the Middle East respiratory syndrome coronavirus (MERS-CoV) epidemic on medical students' perception and found that all of these students experienced stress [21].

Another study conducted in Egypt assessed the psychological burden of (2019-nCoV) through the Impact of Event Scale-Revised (IES-R) score, where more than half of the participants (52%) showed moderate and severe psychological impact, whilst 23.9% reported mild impact [22].

We found that anxiety appeared to motivate preventive behavior; those people with a moderate level of anxiety were most likely to take comprehensive precautionary measures against the infection. The outbreak itself and the control measures may lead to widespread fear and panic, especially stigmatization and social isolation of confirmed patients, survivors, and relations, who may escalate into further negative psychological reactions.

Our sample revealed that the internet (73.2%) was the most common source of primary health information for the general public during (2019-nCoV) epidemic, followed by official health authority statements through television. Despite this, the perceived availability of resources of information was not found to affect knowledge and only 20.7% of respondents were able to correctly answer all three questions. The results revealed that there was no correlation between the public's knowledge about (2019-nCoV) and the adoption of precautionary measures to prevent the spread of infection. These findings confirm that knowledge of a disease is not sufficient to trigger preventive action.

As the (2019-nCoV) epidemic continues to spread, healthcare professionals were uncertain about how to control the epidemic. Consequently, assessing public opinion of authorities' crisis management in our survey in Egypt was highly positive and distinctive. However, the quality of official information of dissemination and acceptance of quarantine regulations wasn't correlated with taking preventive measures. This may be related to their high positive rating.

Our findings revealed that older, women and more educated people brought about the greatest uptake of preventive measures. In a study by Wong et al. about MERS-CoV outbreak perceptions of risk and stress evaluation among health care personnel; females were found to have significantly more fear of contracting the infection compared to males in terms of personal protection and social avoidance [23]. In another study, undergraduate female students reported more worries and exaggerated responses [24].

Likewise, a study in Middle East and North Africa (MENA) region assessed the psychological impact of (2019-nCoV) using the Impact of Event Scale-Revised (IES-R) and found that females and participants aged 26–35 years were more likely to have higher stress scores [25]. The biological, social and cognitive processes underlying gender differences in the susceptibility to psychological disorders have not yet been fully understood. However, the fluctuations in ovarian hormone levels may be responsible for altered sensitivity to emotional stimuli among women [26],[27].

As anticipated, younger, less educated males were least likely to adopt appropriate preventive measures in protecting themselves. This underlines the importance of health promotion messages for in attempt to raise the level of protective precautions undertaken by this vulnerable subgroup.

In a study of elderly males and females in Egypt, the authors found that females who had lived in rural areas and were living in poor residences were more likely to be disabled than women in better circumstances. For males, only illiteracy was associated with disablement. This was attributed to the fact that literacy is much more prevalent among men, and those who are illiterate are, therefore, more likely to be poor. For women, living in rural areas is associated with having large families and a tendency to rely on traditional healers for births and medical needs. They are, thus, more exposed than men to poor medical care for reproductive healthcare and, consequently, more at risk of infection [28].

Thus, this subgroup of the population that are at a greater risk of experiencing fear and anxiety will need special attention from public health professionals. Several measures to deal with the mental and psychological stress during the outbreak response have been published by WHO, Centers for Disease Control and Prevention (CDC), and United Nations International Children's Fund (UNICEF) [29]–[31]. Among these measures, which we think are best relevant to the situation in Egypt. Public education and creating awareness of the severity of the virus is very important through media platforms to stop the spread of the disease. Social media and other communication methods may be a source of misinformation, which may increase the level of fear and negative reactions. Therefore, accurate data and information should be carefully selected. It is also important to amplify positive and hopeful stories of people who recovered from the disease. Providing emotional support from family, colleagues, and managers to the affected people during different stages of isolation/treatment can help them overcome the psychological impact.

The present study had some limitations: i) The results were obtained based on self-report information and self-administered tools, and may therefore suffer from bias. ii) In addition, the questions exploring safety behaviors and precautionary measures based on the author's experience and previously published studies on pandemics iii) The study's cross-sectional design did not permit the elucidation of causal relationships. iv) Although an online survey may provide a large amount of data collection within a short period which may be equivalent to paper-and-pencil data collection, it remains subject to criticism concerning data quality; v) The online survey may be representative for only internet community; as older, and less educated groups may not be presented.

## 5. Conclusions

A pandemic is a public health emergency; they have an impact on mental health. This study investigated the psychological impact of (2019-nCoV) in Egypt during the initial phase of the pandemic, as well as the complex associations between fear of (2019-nCoV) symptoms, anxiety symptoms, as well as compliance with safety behaviors.

Based on this study's results, 48% of respondents reported moderate anxiety symptoms. We found that those people with a moderate level of anxiety as well as older, women and more educated were most likely to take comprehensive precautionary measures against the infection. Whereas, those risk-taking individuals e.g., young males may require adjustment of their attitude to optimize self-protection measures against (2019-nCoV) infection and community spread. Therefore, we recommend health promotion campaigns and communication techniques to be developed and rigorously evaluated their effectiveness for public health. Future studies, focusing on groups that are more vulnerable to pandemic-related stress, such as the people with medical conditions, psychiatric patients and healthcare professionals, are also of great importance. Furthermore, long-term consequences of (2019-nCoV) on mental health remains to be evaluated.
