# Supplementary material for: Assessment of community psycho-behavioral responses during the outbreak of novel coronavirus (2019-nCoV): a cross-sectional study
Source: AIMS Public Health. 2021 Nov 9;9(1):26–40. doi: 10.3934/publichealth.2022003 (PMC8755960; doi:10.3934/publichealth.2022003)
Supplement: Supplementary file 1 [file publichealth-09-01-003-s001.pdf]

AIMS *Public Health*

AIMS Public Health, 9(1): 26–40.
DOI:10.3934/publichealth.2022003
Received: 09 September 2021
Accepted: 01 November 2021
Published: 09 November 2021

http://www.aimspress.com/journal/aimsph

Research article

Assessment of community psycho-behavioral responses during the outbreak of novel coronavirus (2019-nCoV): a cross-sectional study

Doaa M Magdy[1,*], Ahmed Metwally[1] and Omar Magdy[2]

[1] Department of Chest Diseases, Faculty of Medicine, Assuit University, Egypt
[2] Medical student Faculty of Medicine, Assuit University, Egypt

* **Correspondence**: Email: doaamagdy_2020@aun.edu.eg; Tel: +2–010–062–610–10.

I-Preventive measures

Questions:	Response
1. “Did you cover your mouth with a paper tissue or handkerchief when sneezing or coughing?”	(a) Always
2. “Did you cover your mouth with your bare hand when sneezing or coughing?”	(b) Most of the time
3. “Did you wash your hands after sneezing, coughing, or cleaning your nose in the past three days?”	(c) Sometimes
	(d) Never
4. “Did you use soap or liquid hand-wash when washing your hands in the past three days?”	(e) Don’t know
5. “Did you wear a mask over your mouth in the past three days?”	
6. “Did you use serving utensils (chopsticks or spoons) for shared food when joining others over the past three days?”	
7. “In the past three days, when touching objects that might possibly carry the (2019-nCoV)) virus [e.g., door handles, buttons in the lifts], did you take preventive measures (e.g., pressing lift buttons with tissue paper)?”	
8. “In the past three days, after touching objects that might possibly carry the (2019-nCoV) virus [e.g., door handles, buttons in the lifts], did you wash your hands as soon as possible?”	

II-Self-Health Perception

a- Physical health complaints

“In the past 2 weeks, have you had any of the following symptoms?”

	Symptoms	Response
1.	Persistent high fever of 38°C	(a) Yes
2.	Sore throat	(b) No
3.	Running nose	(c) Don't know
4.	Having aches all over the body	
5.	Headache	
6.	Cough	
7.	Rapid breathing	

b- Generalized Anxiety Disorder Questionnaire (GAD-7)

Over the last 2 weeks, how often have you been bothered by any of the following problems?	Response
1. Feeling nervous, anxious or on edge?	a. Not at all
2. Not being able to stop or control worrying	b. Several days
3. Worrying too much about different things?	c. More than half the days
4. Trouble relaxing?	d. Nearly every day
5. Being so restless that it is hard to sit still?	
6. Becoming easily annoyed or irritable?	
**7.** Feeling afraid as if something awful might happen?	

c- Attitude to public fear score

	Response
1. Likelihood of contracting during (2019-nCoV) outbreak	a) Very likely
	b) Somewhat likely
	c) Not very likely
	d) Not likely at all
	e) Don't know
2. Likelihood of surviving if infected	a) Very likely
	b) Somewhat likely
	c) Not very likely
	d) Not likely at all
	e) Don't know

III-Knowledge about (2019-nCoV)

a- Routes of transmission

	Response
1. In your opinion, is (2019-nCoV) transmitted through saliva droplet transmission?"	(a) Agree
2. In your opinion, is (2019-nCoV) transmitted through airborne transmission?"	(b) Disagree
3. In your opinion, is (2019-nCoV) transmitted through hand-contact transmission?"	(c) Don't know

IV-Appraisal of Crisis Management Responses:

a- Official Crisis Information

	Response
1. "With regards to the distribution of information by the health authorities to the public in Egypt, do you agree or disagree that it has generally been accurate?"	a) Strongly disagree
2. "With regards to the distribution of information by the health authorities to the public in Egypt, do you agree or disagree that it has generally been clear?"	b) Disagree
3. "With regards to the distribution of information by the health authorities to the public in Egypt, do you agree or disagree that it has generally been sufficient?"	c) Not sure, but probably disagree
4. "With regards to the distribution of information by the health authorities to the public in Egypt, do you agree or disagree that it has generally been timely?"	d) Not sure, but probably agree
5. "With regards to the distribution of information by the health authorities to the public in Egypt, do you agree or disagree that it has generally been trustworthy?"	e) Agree<br>f) Strongly agree

	Response
Acceptance of Regulations<br>"If you did not develop symptoms of (2019-nCoV) after having nonclose contact with someone diagnosed with (2019-nCoV), would you agree to be quarantined for 10 days?"	(a) Agree<br>(b) Don't agree<br>(c) Don't know

AIMS Press ©2022 the Author(s), licensee AIMS Press. This is an open access article distributed under the terms of the Creative Commons Attribution License (http://creativecommons.org/licenses/by/4.0)
